# U.S. Military veterans and the opioid overdose crisis: a review of risk factors and prevention efforts

**DOI:** 10.1080/07853890.2022.2092896

**Published:** 2022-07-06

**Authors:** Alex S. Bennett, Honoria Guarino, Peter C. Britton, Dan O’Brien-Mazza, Stephanie H. Cook, Franklin Taveras, Juan Cortez, Luther Elliott

**Affiliations:** a School of Global Public Health, New York University, New York, NY, USA; b Center for Drug Use and HIV/HCV Research (cduhr.org), New York, NY, USA; c CUNY Graduate School of Public Health & Health Policy, New York, NY, USA; d VA Center for Excellence, University of Rochester, Rochester, NY, USA; e Veterans Flight Plans Consulting, Atlanta, GA, USA; f OnPoint, New York Harm Reduction Educators/Washington Heights Corner Project, New York, NY, USA

**Keywords:** Opioids, U.S. military veterans, overdose, pain management

## Abstract

U.S. military veterans have been heavily impacted by the opioid overdose crisis, with drug overdose mortality rates increasing by 53% from 2010–2019. Risk for overdose among veterans is complex and influenced by ongoing interaction among physiological/biological, psychological, and socio-structural factors. A thorough understanding of opioid-related overdose among veterans, one that goes beyond simple pharmacological determinism, must examine the interplay of pain, pain treatment, and stress, as well as psychological and social experiences—before, during, and after military service. Comprehensive efforts to tackle the overdose crisis among veterans require interventions that address each of these dimensions. Promising interventions include widespread naloxone distribution and increased provision of low-threshold wrap-around services, including medications for opioid use disorder (MOUD) and holistic/complementary approaches. Interventions that are delivered by peers – individuals who share key experiential or sociodemographic characteristics with the population being served – may be ideally suited to address many of the barriers to opioid-related risk mitigation common among veterans. Community care models could be beneficial for the large proportion of veterans who are not connected to the Veterans Health Administration and for veterans who, for various reasons including mental health problems and the avoidance of stigma, are socially isolated or reluctant to use traditional substance use services. Interventions need to be tailored in such a way that they reach those more socially isolated veterans who may not have access to naloxone or the social support to help them in overdose situations. It is important to incorporate the perspectives and voices of veterans with lived experience of substance use into the design and implementation of new overdose prevention resources and strategies to meet the needs of this population.

**Key messages**
1. U.S. military veterans have been heavily impacted by the opioid overdose crisis, with drug overdose mortality rates increasing by 53% from 2010–2019.
2. The risks for overdose that veterans face need to be understood as resulting from an ongoing interaction among biological/physiological, psychological, and social/structural factors.
3. Addressing drug overdose in the veteran population requires accessible and non-judgemental, low threshold, wraparound, and holistic solutions that recognise the complex aetiology of overdose risk for veterans.

## 1. Introduction

The U.S. remains in a decades-long public health crisis involving opioid-related morbidity and mortality [1,2], and military veterans continue to be heavily impacted [3], with rates of overdose mortality among veterans increasing by over 50 percent between 2010 and 2019 [4–7]. In what follows, we consider the current overdose crisis among military veterans, charting the alarming increases in overdose morbidity and mortality that have impacted the veteran population in the past 30 years. It is undeniable that combat-related injury and liberal opioid prescribing can create an iatrogenic pathway to opioid dependence. However, a more comprehensive understanding of opioid use disorder (OUD) and related harms, including overdose, among this population requires a more holistic approach. That approach needs to attend to physiological, psychological and social factors from a multidimensional perspective. In this article, we review epidemiological, public health, and social scientific research literatures to advance a biopsychosocial perspective on overdose risk among veterans. A focus on physical pain alone, for example, results in a simple narrative that pain is a precursor of opioid use and then of mortality risk. For veterans, this narrative of service-related injury and subsequent pain experiences may ultimately explain only a small proportion of OUD and other risky opioid use. A more complete understanding of opioid-related overdose among veterans must examine the often complex and idiosyncratic sequencing of exposures to pain, stress, and pain treatment within an individual life course. So too must psychosocial experiences be highlighted such that adverse childhood experiences, including abuse, poverty and socioeconomic exclusion, are considered alongside military experience and the often challenging process of reintegration after military service. Accordingly, in this review article, we seek to draw upon a wide range of scholarship and research that centres the interplay of physiology, psychology, and sociology to highlight the multiple avenues towards remediation of opioid-related mortality among the veteran population in the U.S.

## 2. Intersecting epidemics – biopsychosocial pain in veterans and the opioid crisis in the U.S

When looking at the current overdose crisis and its impact on veterans, it is important to situate the current crisis within a broader socio-historical context [8,9]. During military conflicts dating back hundreds of years, U.S. service members have used various substances to enhance performance in combat and to manage various forms of physiological, psychological and social pain [10]. At times, the use of various substances has led to negative health outcomes like iatrogenic addiction and overdose [11,12]. However, it was not until the last decades of the twentieth century that the contours of the opioid overdose crisis as it now appears emerged, impacting veterans among other groups.

Specifically, this current crisis needs to be understood in the context of the relatively recent history of deindustrialisation, suburbanisation, and widening social and structural inequality that shaped the post-WWII landscape in the U.S. [11,13]. Economic restructuring and suburbanisation resulted in high levels of concentrated poverty and unemployment in many communities. Ultimately, this was associated with a significant increase in “deaths of despair,” a term recently coined by two economists [14]. The term refers to deaths from drug overdose, alcohol related problems and suicide, which have risen in the past two decades and reduced the overall life expectancy of U.S. adults. Some of the highest overdose rates have occurred in states with high levels of poverty and deindustrialisation, conditions that also drove many people to enlist in the military as jobs in traditional industries were disappearing [15]. Thus, many already vulnerable young people from disadvantaged backgrounds enlisted in the military. Quite frequently they incurred additional vulnerabilities for problematic opioid use and overdose through their military service experiences, then returned to these same communities with limited economic opportunities after their separation from the military [16].

In the early 1990s, when U.S. soldiers were returning from the first Gulf War conflict, Operation Desert Storm, an influential and lasting convergence of pharmacological, medical, social, economic and technological factors helped catalyse what would become an unprecedented rise in opioid-involved overdose [15,17]. Over the next three decades, the U.S. witnessed sustained, year-over-year increases in overdose deaths, culminating most recently in over 100,000 lives lost during a single 12-month period ending in April 2021 [18]. In the early years of this era, doctors were increasingly prescribing prescription opioids (POs) as a long-term treatment for patients with chronic pain, including many military veterans. New, high-potency and long-acting opioid formulations had entered the market under brand names including OxyContin (in 1996) and Opana (in 2006). Through pharmaceutical industry marketing efforts and the claims of several empirically limited early research observations, opioid pain medications were established as a relatively safe and non-addictive “gold standard” treatment for non-cancer pain [19,20]. Moreover, with Desert Storm and, shortly after the turn of the twenty-first century, Operation Enduring Freedom (OEF), more soldiers were surviving battlefield injuries and dealing with service-related injuries, increasingly through the use of opioid-based pain medications. Major innovations in battlefield medicine and protective equipment resulted in a higher survival rate for critically injured soldiers, injuries that would have been fatal in previous conflicts [21]. One consequence is that chronic pain has been widespread among recent generations of veterans: 48% of OEF/Operation Iraqi Freedom (OIF)/Operation New Dawn (OND) veterans of the second Gulf War entering the Department of Veterans Affairs’ Veterans Health Administration medical system (henceforth, “the VA”) were diagnosed with a chronic pain disorder within one year [22,23]. Some pain patients, including military veterans, who had been prescribed and used POs over extended periods developed not only physiological dependence on opioids, but also iatrogenic opioid use disorder, characterised by compulsive use of opioids despite negative impacts on multiple life domains, including increased risk of premature death [24,25].

### 2.1. Pain as the “fifth vital sign”

Abundant research, journalistic investigations, and court cases have now documented the role played by Big Pharma and some medical professionals in creating the opioid overdose crisis that emerged in the 1990s. In particular, the industry capitalised on the American Pain Society’s 1995 designation of pain as the “Fifth Vital Sign” as a rationale for the aggressive marketing and widespread prescription of opioids [20]. In 1998, the Veterans Health Administration (VHA) formally established the improved treatment of acute and chronic pain as a national priority for its network of facilities with the launch of its National Pain Management Strategy (VHA Directive 2009-053) which mandated routine screening for and prompt treatment of pain. During this period, the pharmaceutical industry consciously targeted veterans experiencing pain as an important consumer base for opioid analgesics, and promoted claims that opioids are efficacious in relieving anxiety and sleep disorders [22], which are also prevalent in veterans [26]. These efforts coincided with the return of many service members from OEF/OIF/OND in Iraq and Afghanistan. Messaging was designed for veterans and disseminated widely, including the 2009 book *Exit Wounds: A Survival Guide to Pain Management for Returning Veterans and their Families* [27], which focussed on opioids as a class of medication that people in pain *deserved* because of claimed improvements in functionality and quality of life. These arguments took advantage of veterans’ concerns about being denied healthcare and socioeconomic support post-service, and leveraged the narrative that those who served the nation were entitled to the best care available [27].

### 2.2. Drug Use during military service

In addition to high rates of service-related injury treated with opioid analgesics in the recent veteran population, medical PO use among active Department of Defense (DOD) personnel also increased – by more than 500% between 2002 (2%) and 2008 (11%). Illicit drug use, including misuse of prescription drugs, rose considerably as well, from 3% in 2002 to 12% in 2008 such that, by 2008, more than 1 in 10 service members was legally or illegally taking opioids [28]. During the same time period, the U.S. Army reported that 25‐35% of soldiers prescribed a PO met DSM-IV criteria for substance dependence while awaiting medical discharge [29], suggesting that pain management was a critical context for the development of opioid dependence even during active duty. Statistics on the rapid escalation of opioid prescribing by military doctors – indicating a quadrupling between 2001 and 2009 [28] – further establish the importance of looking at in-service iatrogenic pathways to OUD. Taken together, these numbers suggest the significance of a simple iatrogenic pathway from exposure to POs in the context of medical treatment for service-related pain to opioid dependence, misuse and/or OUD. However, the observed patterns could in fact be the product of a number of different trajectories in opioid access and use. For example, research has found that, for at least some veterans, substance use disorders predated military service, as did other mental health disorders [24,30]. Other research that complicates the simple iatrogenic pathway model has found that some veterans, especially among the cohorts born prior to 1970 and after 1984, used heroin prior to initiating medical PO use and prior to military enlistment [31].

### 2.3. Prescription opioids, substitutions, and transitions to heroin

About fifteen years into the new millennium, a spate of new measures intended to curb liberal PO prescribing and PO diversion was implemented in the face of mounting evidence of opioid-related harms [20]. Efforts that ranged from revamped prescribing guidelines for physicians, to prescription drug monitoring programs (PDMPs) designed to thwart “doctor-shopping,” to the pursuit of civil and criminal penalties for PO suppliers, were instituted across the country and quickly began to constrict the supply of POs [8,17]. Policy shifts within the VA likewise reined in dispensing of opioid pain medications [32]. More specifically, in 2016, the Centres for Disease Control and Prevention (CDC) issued new guidelines for the use of POs in the treatment of chronic pain that advocated for an extremely judicious, tightly controlled and monitored approach to prescribing POs, particularly when used on a long-term basis or in high-dosage formulations [33]. The CDC guidelines were reflected in the 2017 VA/DoD Opioid Therapy Clinical Practice Guidelines which likewise emphasised the importance of risk assessment and the use of risk mitigation strategies when initiating and maintaining chronic pain patients on opioid therapy [32]. These types of supply-side measures may have inadvertently increased the likelihood of riskier forms of opioid use among veterans (and others), as some veterans turned to diverted POs or heroin to manage their pain and stave off opioid withdrawal [34,35]. Moreover, through the 1990s and 2000s, prices per milligram for diverted POs surged, and high street prices combined with diminished access led some people who use opioids to turn to heroin or initiate insufflation and/or injection use of POs as cost-saving measures [16,36–38]. In this context of shifting policies and market conditions that limited access to POs, some veterans turned to other, riskier sources of opioids to manage their pain when they were no longer able to obtain opioid pain medication from their healthcare provider [39].

### 2.4. The recent epidemiology of overdose among Veterans - Polysubstance use and comorbid substance use disorders

The overdose rate among veterans steadily climbed beginning in the late 1990s due to many of the factors discussed above, a trend that has continued largely unabated according to recent published reports [6,7]. Among the roughly 30% (6.1 million of 20.3 million) of veterans who use VA services, the prevalence of overdose deaths from non-synthetic opioids roughly doubled between 2001 and 2009 [5], and overdose deaths in this population have continued to rise dramatically, showing a 65 percent increase from 2010 to 2016 alone [39]. By about 2010, heroin-related overdoses were becoming more common in the U.S. Starting in 2013, illicitly manufactured fentanyl, an array of synthetic analogues of fentanyl, some of them up to 100 times stronger than morphine, was increasingly present in the illegal drug supply [40–42]. This laid the groundwork for what one researcher has called the “triple wave epidemic” in reference to the shift in primary drivers for overdose from POs to heroin and, most recently, illicitly manufactured fentanyl analogues [43].

At the same time, while opioid-involved overdoses continued to climb, data demonstrated that the majority of drug-related fatalities (in veterans and non-veterans alike) involved multiple substances, especially benzodiazepines, alcohol, and more recently, stimulants [18]. A retrospective national cohort study of veterans diagnosed with OUD who were receiving care from the VA in 2017 (*n* = 65, 741) found that the majority appeared to have at least one substance use disorder in addition to OUD and many had multiple substance use disorders (e.g. methamphetamine, benzodiazepines) [44]. In recent years, evidence shows that stimulant use is on the rise among veterans, as it is in the general US population. In a retrospective cohort study of patients who died from stimulant-involved overdose between 2012 and 2018, the rate of deaths from stimulant-related overdose among veterans was three times higher in 2018 than 2012 [39,45]. Another study found that between 2010 and 2019, drug overdose mortality increased by 333.4% for overdoses involving stimulants and by 93.4% for overdoses involving opioids [7].

For many veterans, military service and recreational culture involves moderate to heavy alcohol use, and the use of alcohol greatly elevates the risk of overdose when combined with opioids [46,47]. Rates of heavy alcohol use, binge drinking, and associated alcohol-related problems have also been shown to be higher among those exposed to combat [48,49]. A recent study using VA records linked to National Death Index data examined trends in alcohol-related overdose mortality between 2012–2018 and found that 2,421 veterans died from an alcohol-involved overdose and as expected, the vast majority of these deaths also involved opioids [50].

## 3. Biopsychosocial perspectives on overdose risk among veterans

Veterans’ opioid use initiation is frequently grounded in service-related injury, which for some, leads to long-term opioid use for chronic pain management [39]. Therefore, even medically supervised, adherent PO use can progress to nonmedical use of POs and/or heroin use, both of which pose heightened overdose risk [51–53]. This is an especially salient problem given the rampant fentanyl contamination of the current illicit opioid supply [40–42]. However, this does not account for the multiple, overlapping biological/physiological, psychological, and social challenges that veterans commonly face – challenges that the literature suggests can function to increase their risk for overdose [16,54]. A useful conceptual scaffolding for these multiple dimensions of overdose risk is the biopsychosocial model (BPS) that helps focus attention on the broad range of non-pharmacological factors that can influence an individual’s or social group’s vulnerability to overdose [55–57]. This BPS framework recognises the potential role of physiological pain (i.e. the *biology* of injury and neurological dysfunction in chronic pain), but also requires consideration of the role of mental health concerns (i.e. the *psychology* of suicidality, depression, and PTSD symptoms such as agitation and hypervigilance) and interpersonal relationships and life events (i.e. the *sociology* of interpersonal and institutional supports and life turning points) [58–60] in synergistically producing behaviours that create risk for overdose.

### 3.1. Mental health

Veterans exhibit high rates of mental health disorders (e.g. depression, anxiety, suicidality, PTSD) [28,61–64], and some may use opioids and/or other substances as a means of coping with emotional pain and trauma [57,65]. While many mental health diagnoses emerge post-military service, evidence also suggests that at least three specific disorders, generalised anxiety, PTSD, and conduct disorders, as well as mental health multi-morbidity, are more common among new soldiers than in the general population [66]. This suggests not only that some mental health disorders among veterans may predate military service, but also that the prevalence of certain mental health disorders may be disproportionately high among the groups of Americans most likely to enter the military. This is relevant to overdose risk, as evidence shows that anxiety and depression can heighten overdose morbidity and mortality risk, particularly when they contribute to solitary use of opioids [67]. Moreover, pharmacological treatment for some mental health problems, such as anxiety and panic disorders, can include benzodiazepine-class anxiolytics which are known to increase overdose risk when used concurrently with opioids [68].

### 3.2. Military Sexual trauma

Military sexual trauma is a case study in how psychosocial trauma can be a precondition for OUD and, by extension, risk for overdose. There is a high prevalence of military sexual trauma among both men and women veterans [69], and a recent study using a large VA patient database found that veterans with a history of military sexual trauma had 50% higher odds of having an OUD diagnosis than those without a history of military sexual trauma, suggesting a practice of self-medication of psychological and emotional pain with opioids [70]. This is especially concerning because military sexual trauma has historically been underreported, suggesting that there is likely much greater need for psychosocial support and other resources for military sexual trauma. [71,72].

### 3.3. Social isolation and lack of supportive relationships

Social isolation is a significant concern among the veteran population [73]. Mental health problems, chronic pain, and ambulatory challenges can contribute to social isolation, and loneliness is associated with opioid use among individuals with OUD [74]. Using opioids alone greatly increases the risk of an overdose fatality, due to the lack of others present who might call emergency services and administer naloxone and/or rescue breathing. The heightened risk to socially isolated veterans who use opioids in solitary contexts is particularly acute in the current era, when illicit fentanyl is highly prevalent in the drug supply. In light of these marked dangers for socially isolated veterans, the presence of supportive social relationships represents a critical protective factor against overdose mortality [75]. People with greater social support may be better positioned to survive a potentially fatal overdose [67,76–79].

### 3.4. Homelessness

Research suggests that opioid-related overdose has become the most common cause of mortality among many homeless populations [80], and homelessness disproportionately impacts veterans. Roughly 9% of the general US population are veterans, but veterans represent over 15% of the homeless population in the U.S. [81]. The vast majority of homeless veterans (97%) report minimal social support [82], effectively increasing their risk of overdose mortality compared to homeless dyads, for example, which can more readily contact 911 or administer naloxone in the event of an overdose. Moreover, mental health challenges and substance use are endemic in the homeless veteran population [83,84]; a national survey of homeless veterans found that 31% of veterans who have been homeless less than two years and 46% of those who have been homeless more than two years were diagnosed with co-occurring mental illness and substance use disorder (SUD) [81]. Recent research has also found that the prevalence of OUD was 12 times higher among homeless veterans as compared to non-homeless veterans [85]. Being homeless and socioeconomically disadvantaged has been found to be associated with SUD and overdose [86–88].

### 3.5. Overdose and suicide

Suicide is a highly pressing issue among veterans, especially those who served in Afghanistan and Iraq, and an unknown proportion of fatal and non-fatal overdose events in veterans may be the result of suicide attempts. Suicide risk has been shown to be higher among OEF/OIF/OND veterans than among the general population [89], and the unique experiences of veterans often require that carefully tailored approaches to therapy be utilised. Much has been written about the link between overdose and suicide [90]. Studies have detailed a strong relationship between substance use, suicidality and overdose in non-veteran populations [76,91,92], and opioid use is associated with suicide in veterans [93]. The role of suicidal ideation in accidental overdose events, as well as overdose risk behaviours such as mixing opioids with benzodiazepines and/or alcohol, is less clear [94], and the distinction between unintentional and intentional overdose can be murky [95]. Clearly, treatment for veterans with OUD needs to be sensitive to the role that risk factors for suicidality, including psychiatric diagnoses and social isolation, can play in motivating substance use behaviours and the role that opioids can play in suicide attempts [16].

### 3.6. Access to and use of health care through the VA

Another significant barrier and potential risk factor for overdose among veterans is a fundamental lack of access to healthcare services and low rates of utilisation of the VA in particular. As noted in the introduction, while fewer than 50% of veterans used at least one VA benefit (such as the GI Bill or GI mortgage), only 6.1 million of the total 20.3 million veteran population (30%) have used VA healthcare. This is concerning on several fronts. First, much of the scientific knowledge about health risks among veterans who use opioids comes from VA samples, which represent less than half of all military veterans [96] at most. Although it is widely believed that non-VA veterans are more likely to be employed and have private insurance, veterans who do not have VA access or choose not to use VA benefits also include many of those most historically disadvantaged and at greatest risk of opioid-related harm, including members of racial and ethnic minorities and those from socioeconomically disadvantaged backgrounds [97–99]. “Other than honorable” discharges for active duty personnel with alcohol or substance use issues have excluded significant numbers of veterans from VA care, though changes since 2017 in Public Health Law 115-141 have resulted in more service-connection for veterans with mental health concerns. VA-based samples, therefore, are likely to underrepresent opioid-related harms among the broader veteran population who do not use the VA, excluding some particularly high-risk populations. At the same time, a small subset of veterans who face complex clinical conditions and often have the most challenging medical needs, are more likely to utilise the VA, potentially skewing the overdose mortality rate upwards due to the high rates of VA utilisation among a population at especially high risk for overdose [39].

## 4. Preventing and responding to overdose among the veteran population

In this final section we describe promising interventions that have been implemented to address overdose among veterans. Following the BPS framework, we review innovative and timely efforts to tackle the overdose crisis among veterans by addressing physiological, psychological, and socio-structural dimensions of risk. Veterans represent a population with a unique potential for being at the vanguard of public health innovation and novel intervention implementation. Despite its early involvement in liberal PO prescribing and iatrogenic dependence, the VA has in more recent years provided naloxone access across its facilities and has implemented innovative peer support programs for veterans with SUD. Nevertheless, in light of the ongoing overdose health crisis and the high numbers of veterans who continue to experience opioid-related morbidity and mortality, additional initiatives are needed. For example, while medication for opioid use disorder (MOUD) is the gold standard of evidence-based OUD treatment, only 38% of persons with OUD across VA healthcare facilities receive MOUD, and barriers such as stigma and discrimination hinder uptake [100]. One recent study found that VA patients with OUD who were older and Black had lower odds of receiving the opioid agonist medication buprenorphine [101], highlighting critical disparities that remain to be addressed.

### 4.1. Peer-led interventions for veterans

Interventions that are delivered by peers who share key experiential or sociodemographic characteristics with the population being served may be ideally suited to address overdose and many of the barriers to opioid-related risk mitigation discussed above. The presence of a peer (in this case, a veteran who has been affected by substance use and has experienced success in managing and reducing substance use or engaging with treatment) can serve to diminish the sense of shame or stigmatisation that veterans may feel when interacting with persons without personal experience of substance use [102,103]. A number of peer-led interventions have shown promise, in particular, peer-based interventions for veterans experiencing homelessness or mental health challenges [83,104–106]. Overall, emerging evidence suggests there are benefits for individuals who receive peer support services as part of their treatment for mental health and/or substance use conditions. Studies have shown the positive benefits of peer support including improvements in treatment engagement and retention, improvements in mental health, decreased substance use, and improvements on quality of life measures [107,108] for individuals who received peer support services as part of their mental health care services. If peer support services were provided to veterans who are experiencing a SUD, and more specifically OUD, there would be a potential for earlier engagement and increased participation in treatment which may ultimately result in fewer deaths caused by opioid-related overdose. The VA has developed a robust peer model that it has been using since the early 2000s, and a growing number of programs at the VA employ peer outreach staff [109,110].

### 4.2. Community Care models

For those veterans who are not connected to the VA, there are very few culturally sensitive and low-threshold interventions available, and with a few exceptions, harm reduction services have not been systematically tailored for and targeted to the veteran population. Community-based care models, often employing peers, and have demonstrated their capacity to effectively engage some of these underserved populations by extending culturally sensitive outreach beyond clinical settings [111,112]. There are an increasing number of veteran service organisations ready to meet this need and provide community-based care for the majority of veterans who do not access the VA. Peer services that work within a harm reduction framework may be best suited to help non-VA-connected veterans who are not interested in or ready for abstinence-based programs. This is consistent with the harm reduction mantra of “meeting people where they are” [113].

### 4.3. The VA and naloxone access for veterans

Perhaps the VA’s biggest accomplishment in the effort to mitigate veterans’ overdose risk is the creation of a system-wide naloxone access and distribution program. In the 2000s, as the opioid crisis was rapidly escalating, public health advocates and community activists (many working at harm reduction agencies) established early methods for distributing naloxone directly to people who use and/or inject drugs, focussing on getting naloxone directly to people who use drugs in community settings [114]. Low-threshold and no-cost naloxone dispensing was then integrated into community-based clinics and treatment programs, as well as several larger health systems and hospitals, the VA being the largest to fully embrace naloxone distribution as an integral component of care for people who use opioids. Now well documented, the VA began implementing naloxone distribution programs in 2014 and subsequently established overdose education and naloxone distribution programs throughout their facilities. This is notable because the VA was able to effectively translate the community-based overdose education and naloxone distribution model into a national healthcare system approach, thereby establishing the largest national naloxone distribution program to people at risk of overdose to date [25,115].

While the VA must be commended for making naloxone widely available in its facilities, next steps should include adopting a comprehensive BPS-informed approach to overdose prevention that attends to the psychological, social, structural and practical barriers faced by the most vulnerable groups of veterans. The VA has embraced multi-modal, non-pharmacological approached to pain care which should help prevent iatrogenic dependency in the course of pain management [116]. In addition to addressing perceived barriers to VA care, when considering how to best address polysubstance- and alcohol-involved overdose, the importance of co-location of services and resources for veterans is critical to ensure veterans in need have access. A recent study found that only 33% of those who died from alcohol-involved overdose received treatment in a substance use disorder clinic in the year preceding death, compared to 65% who were seen in mental health and 86% in primary care [50]. Moreover, some veterans use the VA infrequently and only for specific services. Low-barrier naloxone access, therefore, needs to be expanded into a broad range of community settings, and regular naloxone carriage strongly encouraged, as research is finding that even though many people own a naloxone kit, they often do not carry it with them on a daily basis for a variety of reasons [117]. It is equally concerning that many people who use opioids, including veterans who use opioids, often do so alone, without naloxone or a person to administer it present [16,25]. Elements of the buddy system, which is widely used in the military [118], could be adapted as a culturally-tailored harm reduction strategy for veterans who use drugs—having a buddy present who is trained in naloxone administration and rescue breathing/CPR could prevent an overdose from becoming fatal [119,120]. While the VA and the many community-based organisations that serve veterans have made significant strides in expanding access to naloxone among veterans who use opioids, efforts must now focus on reaching more socially isolated veterans who may not frequent the service settings where naloxone is typically distributed or have the social support to help them in overdose situations.

Additionally, it is critically important that opioid safety messaging and naloxone reach those veterans who use opioids and/or other drugs irregularly – e.g. on weekends or holidays –who may have a false sense of safety about their risk for overdose. Given current illicit drug market conditions, even casual users of illicit drugs are at very real risk of overdose, as reports of fentanyl contamination of cocaine and counterfeit pills, including POs and benzodiazepines, are increasingly frequent throughout the U.S. In light of this risk, opioid safety messaging must also emphasise the importance of using fentanyl test strips (where legal) or other available drug checking technologies to test all illicit drugs for the presence of fentanyl before consuming them. Whenever possible, messaging should also emphasise the importance of using drugs in the presence of a trusted friend who could administer naloxone and call 911 in an overdose event.

To help optimise feasibility, acceptability and effectiveness, these community care and peer outreach models of overdose prevention should be adapted for the local implementation context and available resources. Where possible, it may be advantageous for overdose prevention initiatives to partner with existing community-based agencies and/or veteran service organisations, co-locating the delivery of harm reduction materials such as naloxone and drug testing technologies (e.g. fentanyl test strips). These promising interventions are not necessarily specific to veterans, though organisations that service veterans are obvious places from which to distribute naloxone and other harm reduction supplies to at-risk groups of veterans. For veterans in rural areas, it is now possible to obtain naloxone and other safer drug use supplies *via* mail delivery from the service Next Distro, an organisation that distributes harm reduction supplies by mail to rural locations across the country [121]; efforts are currently underway to extend the reach of this service by expanding mail delivery platforms [122].

### 4.4. Learning from veterans

Perhaps the best suggestions for how to prevent overdose among veterans are provided by veterans themselves who have lived experience of opioid and other drug use. Veterans we have interviewed in our research have emphasised the need for additional low-threshold, peer-delivered services to address many of the ongoing substance-related challenges they and other veterans face [3,16]. Ensuring that veterans can readily access a continuum of services and support from veterans' service organisations at every stage of their military/veteran career, from pre-deployment to deployment, post-deployment, and post-separation from the military, was seen as critical [3]. There was also much interest in expanding access to MOUD, particularly buprenorphine and methadone, as well as complementary and Eastern medicine approaches to enhance well-being (e.g. acupuncture) [16].

Veterans with whom we have worked have also spoken directly to overdose and affirmed their central goal of keeping their fellow veterans alive and safe from disease and accidental and preventable causes of death. Helping to equip veterans with the resources to actualise their life goals may be the best treatment of all. Concretely, veterans suggested expanding short- and long-term MOUD treatments and making harm reduction resources and supplies, such as naloxone and fentanyl test strips, more readily available [123]. A disinclination to seek medical treatment is something that is prevalent in military culture and is often carried over into post-separation life. A fear of appearing weak and vulnerable, reinforced by the emphasis in military culture on masculinity and stoicism [124], leads some veterans to be hesitant to seek medical, mental health and substance use treatment, and even to acknowledge that they have a problem [125]. Furthermore, some veterans have developed a lack of trust of the VA [65]. Others fear they will lose their benefits if they report having a substance use problem [126] or disclose their use of medical marijuana, for example. As noted above, these unique barriers have led some veterans to self-medicate, as in the use of diverted POs, without the safety of medical oversight [3].

Veterans also expressed concern that their potential advancement within the military would be limited if they accepted treatment such as MOUD [24]. For example, veterans often look to police or fire departments for employment post-separation. However, many police departments in particular have screening protocols for mental health and substance use issues that can disqualify a veteran from consideration, especially if they have a diagnosis of PTSD in their files. Veterans need to be wary and protected in terms of what they report if they want certain jobs and they are often aware very early in their service both the benefits and pitfalls that are conferred with a mental health diagnosis [127]. In light of this, community-based organisations need to know how to work not just with stigma but with real, economically limiting policies that disproportionately affect veterans. The VA might also work to ensure veterans that treatment for drug use will remain confidential so they do not feel the need to report to potential employers.

## 5. Concluding thoughts

Despite the efforts detailed above to curb opioid and other substance-related mortality among veterans, the opioid overdose public health crisis persists, with the highest number of overdose fatalities ever reported during 2020–2021 among the general population. Many veterans face difficult challenges combining problems of pain management, substance use, service-related mental and physical health problems, and the day-to-day challenges associated with housing, employment and relationships, all of which can heighten risk for drug overdose. As we have highlighted in this review, veterans’ drug-related problems, and in particular, overdose risk, must be understood in light of their distinct and evolving life contexts and situations – as the product of ongoing interactions across physiological, psychological social, and structural domains. Pain management needs must be understood alongside the larger complex of issues veterans face over the civilian/military/veteran career, acknowledging the physiological, psychological, social and structural factors at play. Addressing and putting resources into veterans’ basic needs, including stable housing, meaningful employment, and holistic, low- threshold, integrated health and healing services are central.

Ultimately, this review suggests the importance of adopting a holistic view in conceptualising overdose risk and in mitigating drug-related harms. Our review of research literature and epidemiological findings demonstrates that veterans’ vulnerability to opioid-related overdose is multifaceted and involves far more than the opioid dependence resulting from long-term management of pain with opioids. Interventions need to address BPS dimensions of risk with multimodal and multidisciplinary approaches to health and well-being.

## Data Availability

Data sharing is not applicable to this article as no new data were created or analysed in this study.

## References

[1] Hedegaard H, Miniño AM, Warner M. Drug overdose deaths in the United States, 1999-2018. 2020. 32487285

[2] Ahmad F, Rossen L, Sutton P. Provisisonal drug overdose death counts. National Center for Health Statistics; 2021.

[3] Bennett AS, Elliott L, Golub A. Veterans’ health and opioid safety-contexts, risks, and outreach implications. Fed Pract. 2015;32(6):4–7. PMC6363306 30766065

[4] Bohnert ASB, Valenstein M, Bair MJ, et al. Association between opioid prescribing patterns and opioid overdose-related deaths. JAMA. 2011;305(13):1315–1321. 21467284 10.1001/jama.2011.370

[5] Bohnert ASB, Ilgen MA, Trafton JA, et al. Trends and regional variation in opioid overdose mortality among veterans health administration patients, fiscal year 2001 to 2009. Clin J Pain. 2014;30(7):605–612. 24281278 10.1097/AJP.0000000000000011

[6] Peltzman T, Ravindran C, Schoen PM, et al. Brief report: opioid-Involved overdose mortality in United States veterans. Am J Addict. 2020;29(4):340–344. 32223045 10.1111/ajad.13027

[7] Begley MR, Ravindran C, Peltzman T, et al. Veteran drug overdose mortality, 2010–2019. Drug Alcohol Depend. 2022;233:109296. 35219064 10.1016/j.drugalcdep.2022.109296

[8] Herzberg D, Guarino H, Mateu-Gelabert P, et al. Recurring epidemics of pharmaceutical drug abuse in america: time for an all-drug strategy. Am J Public Health. 2016;106(3):408–410. 26794163 10.2105/AJPH.2015.302982 PMC4762211

[9] Musto DF. The American disease: origins of narcotic control. New York: Oxford; 1987.

[10] Bergen-Cico DK. War and drugs: the role of military conflict in the development of substance abuse. Boulder, Co: Paradigm; 2011.

[11] Courtwright DT. Dark paradise: a history of opiate addiction in America. Enl. ed. Cambridge (MA): Harvard University Press; 2001. xiii, 326. p.

[12] Courtwright DT, Joseph H, Des Jarlais D. Addicts who survived: an oral history of narcotic use in America, 1923-1965. 1st ed. Knoxville: University of Tennessee Press; 1989. xvi, 399. p.

[13] Jackson KT. Crabgrass frontier: the suburbanization of the United States. Oxford University Press; 1987.

[14] Case A, Deaton A. Deaths of despair and the future of capitalism. Princeton University Press; 2020.

[15] Quinones S. Dreamland: the true tale of America's opiate epidemic. Bloomsbury Publishing USA; 2015.

[16] Bennett AS, Elliott L, Golub A, et al. Opioid-Involved overdose among male Afghanistan/Iraq-Era U.S. Military veterans: a multidimensional perspective. Subst Use Misuse. 2017;52(13):1701–1711. 28622067 10.1080/10826084.2017.1306563 PMC5628147

[17] Dasgupta N, Beletsky L, Ciccarone D. Opioid crisis: no easy fix to its social and economic determinants. Am J Public Health. 2018;108(2):182–186. 29267060 10.2105/AJPH.2017.304187 PMC5846593

[18] CDC. Provisional drug overdose death counts. 2021.

[19] Von Korff MR, Franklin G. Responding to America’s iatrogenic epidemic of prescription opioid addiction and overdose. Med Care. 2016;54(5):426–429. 27075900 10.1097/MLR.0000000000000537

[20] Kolodny A, Courtwright DT, Hwang CS, et al. The prescription opioid and heroin crisis: a public health approach to an epidemic of addiction. Annu Rev Public Health. 2015;36(1):559–574. 25581144 10.1146/annurev-publhealth-031914-122957

[21] Institute of Medicine Committee on Advancing Pain Research. Relieving pain in America: a blueprint for transforming prevention, care, education, and research. National Academies Press; 2011. 22553896

[22] Seal KH, Shi Y, Cohen G, et al. Association of mental health disorders with prescription opioids and high-risk opioid use in US veterans of Iraq and Afghanistan. J Am Med Assoc. 2012;307(9):940–947. 10.1001/jama.2012.234 22396516

[23] Bray RM, Olmsted KR, Williams J. Misuse of prescription pain medications in US active duty service members, in pain Syndromes - From recruitment to returning troops: wounds of war IV. B.K. Wiederhold, editor. Amsterdam: IOS Press; 2012. p. 3–16.

[24] Bennett AS, Elliott L, Golub A. Opioid and other substance misuse, overdose risk, and the potential for prevention among a sample of OEF/OIF veterans in New York city. Subst Use Misuse. 2013;48(10):894–907. 23869461 10.3109/10826084.2013.796991 PMC3789237

[25] Bennett A, Elliott L. Naloxone's role in the national opioid crisis-past struggles, current efforts, and future opportunities. Transl Res. 2021;234:43–57. 33684591 10.1016/j.trsl.2021.03.001 PMC8327685

[26] Rice VJB, Schroeder PJ. Self-Reported sleep, anxiety, and cognitive performance in a sample of U.S. Military active duty and veterans. Military Medicine. 2019;184(Suppl_1):488–497. 30901421 10.1093/milmed/usy323

[27] McGinnis D, Braun SR. Exit wounds: a survival guide to pain management for returning veterans and their families. Waterford Life Science; 2009.

[28] Institute of Medicine, Substance use disorders in the U.S. armed forces. Washington (DC): The National Academies Press; 2012.

[29] U.S. Army, Army 2020: generating health & discipline in the force ahead of the strategic reset. Headquarters, Department of the Army; 2012.

[30] Nock MK, Ursano RJ, Heeringa SG, Army STARRS Collaborators, et al. Mental disorders, comorbidity, and pre-enlistment suicidal behavior among new soldiers in the U.S. Army: results from the army study to assess risk and resilience in servicemembers (army STARRS). Suicide Life Threat Behav. 2015;45(5):588–599. 25622860 10.1111/sltb.12153 PMC4515394

[31] Elliott L, Golub A, Bennett A. Opioid use initiation, progression, and motivations among OEF/OIF/OND-Era veterans in New York city: an Age-Period-Cohort analysis. Mil Behav Health. 2018;6(1):75–81. 29545974 10.1080/21635781.2017.1343163 PMC5847302

[32] Rosenberg JM, Bilka BM, Wilson SM, et al. Opioid therapy for chronic pain: overview of the 2017 US department of veterans affairs and US department of defense clinical practice guideline. Pain Med. 2018;19(5):928–941. 29025128 10.1093/pm/pnx203

[33] Dowell D, Haegerich TM, Chou R. CDC guideline for prescribing opioids for chronic pain—United States, 2016. JAMA. 2016;315(15):1624–1645. 26977696 10.1001/jama.2016.1464 PMC6390846

[34] Harocopos A, Goldsamt LA, Kobrak P, et al. New injectors and the social context of injection initiation. Int J Drug Policy. 2009;20(4):317–323. 18790623 10.1016/j.drugpo.2008.06.003 PMC2706152

[35] Neaigus A, Gyarmathy VA, Miller M, et al. Transitions to injecting drug use among noninjecting heroin users: social network influence and individual susceptibility. JAIDS J Acq Imm Defic Syndr. 2006;41(4):493–503. 10.1097/01.qai.0000186391.49205.3b 16652059

[36] Inciardi JA, Surratt HL, Cicero TJ, et al. Prescription opioid abuse and diversion in an urban community: the results of an ultrarapid assessment. Pain Med. 2009;10(3):537–548. 19416440 10.1111/j.1526-4637.2009.00603.x PMC2719877

[37] Sherman SG, Smith L, Laney G, et al. Social influences on the transition to injection drug use among young heroin sniffers: a qualitative analysis. Inter J Drug Policy. 2002;13(2):113–120.

[38] Lankenau SE, Walley A. Opioids and deaths. N Engl J Med. 2011;364(7):686. 10.1056/NEJMc1014490 21323558

[39] Lin LA, Peltzman T, McCarthy JF, et al. Changing trends in opioid overdose deaths and prescription opioid receipt among veterans. Am J Prev Med. 2019;57(1):106–110. 31128955 10.1016/j.amepre.2019.01.016

[40] Ciccarone D. Fentanyl in the US heroin supply: a rapidly changing risk environment. Int J Drug Policy. 2017;46:107–111. 28735776 10.1016/j.drugpo.2017.06.010 PMC5742018

[41] Armenian P, Vo KT, Barr-Walker J, et al. Fentanyl, fentanyl analogs and novel synthetic opioids: a comprehensive review. Neuropharmacology. 2018;134(Pt A):121–132. 29042317 10.1016/j.neuropharm.2017.10.016

[42] Suzuki J, El-Haddad S. A review: fentanyl and non-pharmaceutical fentanyls. Drug Alcohol Depend. 2017;171:107–116. 28068563 10.1016/j.drugalcdep.2016.11.033

[43] Ciccarone D. The triple wave epidemic: supply and demand drivers of the US opioid overdose crisis. Int J Drug Policy. 2019;71(February):183–188. 30718120 10.1016/j.drugpo.2019.01.010 PMC6675668

[44] Lin LA, Bohnert ASB, Blow FC, et al. Polysubstance use and association with opioid use disorder treatment in the US veterans health administration. Addiction. 2021;116(1):96–104. 32428386 10.1111/add.15116

[45] Coughlin LN, et al. Patient characteristics and treatment utilization in fatal stimulant-involved overdoses in the United States veterans health administration. Addiction. 2022;117(4):998–1008. 34648209 10.1111/add.15714

[46] Besse K, et al. How soldiers perceive the drinking environment in communities near military installations. J Alcohol Drug Educ. 2018;62(1):71–90.

[47] Bray RM, Brown JM, Williams J. Trends in binge and heavy drinking, alcohol-related problems, and combat exposure in the US military. Subst Use Misuse. 2013;48(10):799–810. 23869454 10.3109/10826084.2013.796990

[48] Jacobson IG, Ryan MAK, Hooper TI, et al. Alcohol use and alcohol-related problems before and after military combat deployment. J Am Med Assoc. 2008;300(6):663–675. 10.1001/jama.300.6.663 PMC2680184 18698065

[49] Bray RM, Hourani LL. Substance use trends among active duty military personnel: findings from the United States department of defense health related behavior surveys, 1980-2005. Addiction. 2007;102(7):1092–1101. 17567397 10.1111/j.1360-0443.2007.01841.x

[50] Lin LA, Bonar EE, Zhang L, et al. Alcohol-involved overdose deaths in US veterans. Drug Alcohol Depend. 2022;230:109196. 34894477 10.1016/j.drugalcdep.2021.109196 PMC8714700

[51] Banerjee G, Edelman EJ, Barry DT, et al. Non‐medical use of prescription opioids is associated with heroin initiation among US veterans: a prospective cohort study. Addiction. 2016;111(11):2021–2031. 27552496 10.1111/add.13491 PMC5056813

[52] Tam CC, Zeng C, Li X. Prescription opioid misuse and its correlates among veterans and military in the United States: a systematic literature review. Drug Alcohol Depend. 2020;216:108311. 33010713 10.1016/j.drugalcdep.2020.108311

[53] Wilder CM, Miller SC, Tiffany E, et al. Risk factors for opioid overdose and awareness of overdose risk among veterans prescribed chronic opioids for addiction or pain. J Addict Dis. 2016;35(1):42–51. 26566771 10.1080/10550887.2016.1107264 PMC4751580

[54] Rigg KK, DeCamp W. Explaining prescription opioid misuse among veterans: a theory-based analysis using structural equation modeling. Military Behav Health. 2014;2(2):210–216.

[55] Engel G. The biopsychosocial model and the education of health professionals. Ann N Y Acad Sci. 1978;310(1 Primary Healt):169–181. 290321 10.1111/j.1749-6632.1978.tb22070.x

[56] Engel GL. The clinical application of the biopsychosocial model. Am J Psychiatry. 1980;137(5):535–544. 7369396 10.1176/ajp.137.5.535

[57] Baria AM, Pangarkar S, Abrams G, et al. Adaption of the biopsychosocial model of chronic noncancer pain in veterans. Pain Med. 2019;20(1):14–27. 29727005 10.1093/pm/pny058

[58] Nagin D, et al. The interrelationship of temporally distinct risk markers and the transition from childhood physical aggression to adolescent violent delinquency, in applied data analytic techniques for turning points research. P. Cohen, editor. New York: Routledge; 2008. p. 17–36.

[59] Cohen, P, editor. Applied data analytic techniques for turning points research. New York: Routledge; 2008.

[60] Elder GH. Military times and turning points in men's lives. Develop Psychol. 1986;22(2):233–245.

[61] Andrews B, Brewin CR, Philpott R, et al. Delayed-onset posttraumatic stress disorder: a systematic review of the evidence. Am J Psychiatry. 2007;164(9):1319–1326. 17728415 10.1176/appi.ajp.2007.06091491

[62] Golub A, Vazan P, Bennett AS, et al. Unmet need for treatment of substance use disorders and serious psychological distress among veterans: a nationwide analysis using the NSDUH. Mil Med. 2013;178(1):107–114. 10.7205/milmed-d-12-00131 PMC3743427 23356128

[63] Kimerling R, Makin-Byrd K, Louzon S, et al. Military sexual trauma and suicide mortality. Am J Prev Med. 2016;50(6):684–691. 26699249 10.1016/j.amepre.2015.10.019

[64] McCarthy JF, Valenstein M, Kim HM, et al. Suicide mortality among patients receiving care in the veterans health administration health system. Am J Epidemiol. 2009;169(8):1033–1038. 19251753 10.1093/aje/kwp010

[65] Schell TL, et al. A needs assessment of New York state veterans: final report to the New York state health foundation. Rand Health Q. 2011;1(1) 10.7249/rhq-01-01-14 PMC4945221 28083170

[66] Rosellini AJ, Heeringa SG, Stein MB, et al. Lifetime prevalence of DSM‐IV mental disorders among new soldiers in the US army: results from the army study to assess risk and resilience in servicemembers (army STARRS. Depress Anxiety. 2015;32(1):13–24.) 25338841 10.1002/da.22316 PMC5111824

[67] Latkin CA, Hua W, Tobin K. Social network correlates of self-reported non-fatal overdose. Drug Alcohol Depend. 2004;73(1):61–67. 14687960 10.1016/j.drugalcdep.2003.09.005

[68] Gressler LE, Martin BC, Hudson TJ, et al. Relationship between concomitant benzodiazepine-opioid use and adverse outcomes among US veterans. Pain. 2018;159(3):451–459. 29189516 10.1097/j.pain.0000000000001111

[69] Wilson LC. The prevalence of military sexual trauma: a Meta-analysis. Trauma Violence Abuse. 2018;19(5):584–597. 30415636 10.1177/1524838016683459

[70] Beckman KL, et al. Associations among military sexual trauma, opioid use disorder, and gender. Am J Prevent Med. 2021;46(3):311–316. 10.1016/j.amepre.2021.08.020 34742619

[71] Maguen S, et al. Gender differences in traumatic experiences and mental health in active duty soldiers redeployed from Iraq and Afghanistan. J Psychiatric Res. 2012;46(3):311–316. 10.1016/j.jpsychires.2011.11.007 22172997

[72] Maguen S, Ren L, Bosch JO, et al. Gender differences in mental health diagnoses among Iraq and Afghanistan veterans enrolled in veterans affairs health care. Am J Public Health. 2010;100(12):2450–2456. 20966380 10.2105/AJPH.2009.166165 PMC2978175

[73] Wilson G, Hill M, Kiernan MD. Loneliness and social isolation of military veterans: systematic narrative review. Occup Med. 2018;68(9):600–609. 10.1093/occmed/kqy160 30551163

[74] McDonagh J, Williams CB, Oldfield BJ, et al. The association of loneliness and non-prescribed opioid use in patients with opioid use disorder. J Addict Med. 2020;14(6):489–493. 32039936 10.1097/ADM.0000000000000629

[75] Bennett AS, Scheidell J, Bowles JM, et al. Naloxone protection, social support, network characteristics, and overdose experiences among a cohort of people who use illicit opioids in New York city. Harm Reduct J. 2022;19(1):1–15. 35246165 10.1186/s12954-022-00604-w PMC8894821

[76] Farrell M, Neeleman J, Griffiths P, et al. Suicide and overdose among opiate addicts. Addiction. 1996;91(3):321–323. 8867195 10.1111/j.1360-0443.1996.tb02282.x

[77] Sherman SG, Gann DS, Tobin KE, et al. The life they save may be mine”: diffusion of overdose prevention information from a city sponsored programme. Int J Drug Policy. 2009;20(2):137–142. 18502635 10.1016/j.drugpo.2008.02.004

[78] Dayton L, Gicquelais RE, Tobin K, et al. More than just availability: who has access and who administers take-home naloxone in Baltimore, MD. PLoS One. 2019;14(11):e0224686. 31697736 10.1371/journal.pone.0224686 PMC6837378

[79] Tobin K, Clyde C, Davey-Rothwell M, et al. Awareness and access to naloxone necessary but not sufficient: examining gaps in the naloxone Cascade. Int J Drug Policy. 2018;59:94–97. 30075401 10.1016/j.drugpo.2018.07.003 PMC6341469

[80] Baggett TP, Hwang SW, O'Connell JJ, et al. Mortality among homeless adults in boston: shifts in causes of death over a 15-year period. JAMA Intern Med. 2013;173(3):189–195. 23318302 10.1001/jamainternmed.2013.1604 PMC3713619

[81] Community Solutions. National survey of homeless veterans in 100,000 homes campaign communities. 2011.

[82] Henry M, Watt R, Rosenthal L, et al. The 2016 annual homeless assessment report (AHAR) to congress, part 1: point-in-Time estimates of homelessness. Washington (DC): U.S. Department of Housing and Urban Development; 2016.

[83] Van Voorhees EE, Resnik L, Johnson E, et al. Posttraumatic stress disorder and interpersonal process in homeless veterans participating in a peer mentoring intervention: associations with program benefit. Psychol Serv. 2019;16(3):463–474. 29369660 10.1037/ser0000231 PMC6060037

[84] Ulmer CS, Van Voorhees E, Germain AE, VA Mid-Atlantic Mental Illness Research Education and Clinical Center Registry Workgroup, et al. A comparison of sleep difficulties among Iraq/Afghanistan theater veterans with and without mental health diagnoses. J Clin Sleep Med. 2015;11(9):995–1005. 26094928 10.5664/jcsm.5012 PMC4543260

[85] Manhapra A, Stefanovics E, Rosenheck R. The association of opioid use disorder and homelessness nationally in the veterans health administration. Drug Alcohol Depend. 2021;223:108714. 33865213 10.1016/j.drugalcdep.2021.108714

[86] Center for Disease Control (CDC) Overdose deaths involving prescription opioids among medicaid enrollees - Washington, 2004-2007. MMWR Morb Mortal Wkly Rep. 2009;58(42):1171–1175. 19875978

[87] Hembree C, et al. The urban built environment and overdose mortality in New York city neighborhoods. 2005. 10.1016/j.healthplace.2004.02.005 15629682

[88] Nandi AK, et al. What explains the association between neighborhood-level income inequality and the risk of fatal overdose in New York city? 2006. 10.1016/j.socscimed.2006.02.001 16597478

[89] Kang HK, Bullman TA. Risk of suicide among US veterans after returning from the Iraq or Afghanistan war zones. JAMA. 2008;300(6):652–653. 18698062 10.1001/jama.300.6.652

[90] Bohnert ASB, Ilgen MA. Understanding links among opioid use, overdose, and suicide. N Engl J Med. 2019;380(1):71–79. 30601750 10.1056/NEJMra1802148

[91] Ravndal E, Vaglum P. Overdoses and suicide attempts: different relations to psychopathology and substance abuse? A 5-year prospective study of drug abusers. Eur Addict Res. 1999;5(2):63–70. 10394035 10.1159/000018967

[92] Hall AJ, Logan JE, Toblin RL, et al. Patterns of abuse among unintentional pharmaceutical overdose fatalities. JAMA. 2008;300(22):2613–2620. 19066381 10.1001/jama.2008.802

[93] Ilgen MA, Bohnert AS, Ganoczy D, et al. Opioid dose and risk of suicide. Pain. 2016;157(5):1079–1084. 26761386 10.1097/j.pain.0000000000000484 PMC4939394

[94] Bohnert ASB, Roeder K, Ilgen MA. Unintentional overdose and suicide among substance users: a review of overlap and risk factors. Drug Alcohol Depend. 2010;110(3):183–192. 20430536 10.1016/j.drugalcdep.2010.03.010

[95] Britton PC, Bohnert ASB, Wines JD, et al. A procedure that differentiates unintentional from intentional overdose in opioid abusers. Addict Behav. 2012;37(1):127–130. 21955872 10.1016/j.addbeh.2011.08.006 PMC4864590

[96] NCVAS. VA Utilization Profile. Washington (DC): U.S. Dept. of Veterans Affairs; 2017. https://www.va.gov/vetdata/docs/Quickfacts/VA_Utilization_Profile.pdf

[97] Pouget ER, Bennett AS, Elliott L, et al. Recent overdose experiences in a community sample of military veterans who use opioids. J Drug Issues. 2017;47(3):479–491. 28845055 10.1177/0022042617701255 PMC5567991

[98] Bennett AS, Golub A, Elliott L. A behavioral typology of opioid overdose risk behaviors among recent veterans in New York city. PLoS One. 2017;12(6):e0179054. 28594892 10.1371/journal.pone.0179054 PMC5464624

[99] Cleland CM, Bennett AS, Elliott L, et al. Between-and within-person associations between opioid overdose risk and depression, suicidal ideation, pain severity, and pain interference. Drug Alcohol Depend. 2020;206:107734. 31775106 10.1016/j.drugalcdep.2019.107734 PMC6980716

[100] Priest KC, McCarty D, Lovejoy TI. Expanding access to medications for opioid use disorder: program and policy approaches from outside the veterans health administration. J Gen Intern Med. 2020;35(Suppl 3):886–890. 33145685 10.1007/s11606-020-06266-3 PMC7609303

[101] Manhapra A, Stefanovics E, Rosenheck R. Initiating opioid agonist treatment for opioid use disorder nationally in the veterans health administration: who gets what? Subst Abus. 2020;41(1):110–120. 31403914 10.1080/08897077.2019.1640831

[102] Malis RS, Roloff ME. The effect of legitimacy and intimacy on peer interventions into alcohol abuse. West J Commun. 2007;71(1):49–68.

[103] Webel AR, Okonsky J, Trompeta J, et al. A systematic review of the effectiveness of peer-based interventions on health-related behaviors in adults. Am J Public Health. 2010;100(2):247–253. 20019321 10.2105/AJPH.2008.149419 PMC2804647

[104] Painter JM, Malte CA, Rubinsky AD, et al. High inpatient utilization among veterans health administration patients with substance-use disorders and co-occurring mental health conditions. Am J Drug Alcohol Abuse. 2018;44(3):386–394. 29095057 10.1080/00952990.2017.1381701 PMC6196078

[105] Malte CA, Cox K, Saxon AJ. Providing intensive addiction/housing case management to homeless veterans enrolled in addictions treatment: a randomized controlled trial. Psychol Addict Behav. 2017;31(3):231–241. 28481614 10.1037/adb0000273

[106] Cox KB, Malte CA, Saxon AJ. Characteristics and service utilization of homeless veterans entering VA substance use treatment. Psychol Serv. 2017;14(2):208–213. 28481606 10.1037/ser0000133

[107] Bassuk EL, Hanson J, Greene RN, et al. Peer-delivered recovery support services for addictions in the United States: a systematic review. J Subst Abuse Treat. 2016;63:1–9. 26882891 10.1016/j.jsat.2016.01.003

[108] Chinman M, George P, Dougherty RH, et al. Peer support services for individuals with serious mental illnesses: assessing the evidence. Psychiatr Serv. 2014;65(4):429–441. 24549400 10.1176/appi.ps.201300244

[109] Barber JA, Rosenheck RA, Armstrong M, et al. Monitoring the dissemination of peer support in the VA healthcare system. Community Ment Health J. 2008;44(6):433–441. 18473174 10.1007/s10597-008-9146-7

[110] Hebert M, Rosenheck R, Drebing C, et al. Integrating peer support initiatives in a large healthcare organization. Psychol Serv. 2008;5(3):216–227.

[111] Ali-Faisal SF, Colella TJ, Medina-Jaudes N, et al. The effectiveness of patient navigation to improve healthcare utilization outcomes: a Meta-analysis of randomized controlled trials. Patient Educ Counsel. 2017;100(3):436–448. 10.1016/j.pec.2016.10.014 27771161

[112] Monteith LL, Wendleton L, Bahraini NH, et al. Together with veterans: VA national strategy alignment and lessons learned from Community-Based suicide prevention for rural veterans. Suicide Life Threat Behav. 2020;50(3):588–600. 31950557 10.1111/sltb.12613

[113] Marlatt GA. Harm reduction: come as you are. Addict Behav. 1996;21(6):779–788. 8904943 10.1016/0306-4603(96)00042-1

[114] Bennett AS, Bell A, Tomedi L, et al. Characteristics of an overdose prevention, response, and naloxone distribution program in pittsburgh and allegheny county, Pennsylvania. J Urban Health. 2011;88(6):1020–1030. 21773877 10.1007/s11524-011-9600-7 PMC3232410

[115] Oliva EM, Christopher MLD, Wells D, Veterans Health Administration Opioid Overdose Education and Naloxone Distribution National Support and Development Workgroup, et al. Opioid overdose education and naloxone distribution: development of the veterans health administration’s national program. J Am Pharm Assoc. 2017;57(2S):S168–S179.e4. 10.1016/j.japh.2017.01.022 28292502

[116] Kligler B, Bair MJ, Banerjea R, et al. Clinical policy recommendations from the VHA state-of-the-Art conference on Non-Pharmacological approaches to chronic musculoskeletal pain. J Gen Intern Med. 2018;33(Suppl 1):16–23. 10.1007/s11606-018-4323-z PMC5902342 29633133

[117] Bennett AS, Freeman R, Des Jarlais DC, et al. Reasons people who use opioids do not accept or carry No-Cost naloxone: qualitative interview study. JMIR Form Res. 2020;4(12):e22411. 33355094 10.2196/22411 PMC7787889

[118] Hall JC. Utilizing social support to conserve the fighting strength: important considerations for military social workers. Smith College Studies Social Work. 2009;79(3-4):335–343.

[119] Heller DI, Stancliff S. Providing naloxone to substance users for secondary administration to reduce overdose mortality in New York city. Public Health Rep. 2007;122(3):393–397. 17518311 10.1177/003335490712200313 PMC1847483

[120] Centers for Disease Control and Prevention. Community-Based opioid overdose prevention programs providing naloxone. Morbidity Mortality Weekly Report. 2012;61(6):101–105. 22337174 PMC4378715

[121] Yang C, Favaro J, Meacham MC. NEXT harm reduction: an online, mail-based naloxone distribution and harm-reduction program. Am J Public Health. 2021;111(4):667–671. 33600254 10.2105/AJPH.2020.306124 PMC7958031

[122] Barnett BS, Wakeman SE, Davis CS, et al. Expanding Mail-Based distribution of Drug-Related harm reduction supplies amid COVID-19 and Beyond. Am J Public Health. 2021;111(6):1013–1017. 33950718 10.2105/AJPH.2021.306228 PMC8101586

[123] Bennett AS. Developing a peer delivery model for naloxone distribution and increasing opioid safety knowledge among veterans. In: Harm reduction coalition. San Diego; 2016.

[124] Sherman N. Stoic warriors: the ancient philosophy behind the military mind. Oxford University Press; 2007.

[125] Lorber W, Garcia HA. Not supposed to feel this: traditional masculinity in psychotherapy with male veterans returning from Afghanistan and Iraq. Psychother Theory Res Pract Train. 2010;47(3):296–305. 10.1037/a0021161 22402087

[126] Bray RM, Olmsted KR, Williams J. Misuse of prescription pain medications in US active duty service members. In: BK Wiederhold, editor. Pain syndromes-from recruitment to returning troops: wounds of war IV. Vol. 91. IOS Press; 2012. p. 3.

[127] Elliott L, Bennett AS, Szott K, et al. Competing constructivisms: the negotiation of PTSD and related stigma among post-9/11 veterans in New York city. Cult Med Psychiatry. 2018;42(4):778–799. 29796782 10.1007/s11013-018-9586-7 PMC6251768

